# Comparative sequence and structure analysis of eIF1A and eIF1AD

**DOI:** 10.1186/s12900-018-0091-6

**Published:** 2018-09-04

**Authors:** Jielin Yu, Assen Marintchev

**Affiliations:** Department of Physiology and Biophysics, Boston University School of Medicine, Boston, MA USA

**Keywords:** eIF1A, eIF1AD, Translation initiation, Protein structure, Sequence homology, Protein-protein interactions

## Abstract

**Background:**

Eukaryotic translation initiation factor 1A (eIF1A) is universally conserved in all organisms. It has multiple functions in translation initiation, including assembly of the ribosomal pre-initiation complexes, mRNA binding, scanning, and ribosomal subunit joining. eIF1A binds directly to the small ribosomal subunit, as well as to several other translation initiation factors. The structure of an eIF1A homolog, the eIF1A domain-containing protein (eIF1AD) was recently determined but its biological functions are unknown. Since eIF1AD has a known structure, as well as a homolog, whose structure and functions have been extensively studied, it is a very attractive target for sequence and structure analysis.

**Results:**

Structure/sequence analysis of eIF1AD found significant conservation in the surfaces corresponding to the ribosome-binding surfaces of its paralog eIF1A, including a nearly invariant surface-exposed tryptophan residue, which plays an important role in the interaction of eIF1A with the ribosome. These results indicate that eIF1AD may bind to the ribosome, similar to its paralog eIF1A, and could have roles in ribosome biogenenesis or regulation of translation. We identified conserved surfaces and sequence motifs in the folded domain as well as the C-terminal tail of eIF1AD, which are likely protein-protein interaction sites. The roles of these regions for eIF1AD function remain to be determined. We have also identified a set of trypanosomatid-specific surface determinants in eIF1A that could be a promising target for development of treatments against these parasites.

**Conclusions:**

The results described here identify regions in eIF1A and eIF1AD that are likely to play major functional roles and are promising therapeutic targets. Our findings and hypotheses will promote new research and help elucidate the functions of eIF1AD.

## Background

Translation initiation in eukaryotes is a multistep process involving over ten eukaryotic translation initiation factors (eIFs) (reviewed in [1–4]).

1. Several eIFs and the initiator Metionyl-tRNA (Met-tRNA_i_) bind to the small ribosomal subunit, forming the pre-initiation complex (PIC). Met-tRNA_i_ is recruited to the ribosome in complex with the GTPase eIF2. eIF2 is an αβγ heterotrimer. eIF2γ is the actual GTPase, responsible for the bulk of the interaction with Met-tRNA_i_, while eIF2α and β play accessory and regulatory roles. The N-terminal tail of eIF2β (eIF2β-NTT) contains three conserved poly-lysine stretches (K-boxes) that mediate binding to eIF5, which is the GTPase-activating protein (GAP) of eIF2.
2. The PIC is recruited to the 5′-end of the mRNA by the Cap-binding complex, composed of eIF4E, 4G, and 4A.
3. The PIC then scans the mRNA until it reaches a start codon in a proper nucleotide context.
4. Start codon recognition (basepairing of the Met-tRNA_i_ anticodon with the start codon) triggers major conformational rearrangements in the PIC, leading to the release of most eIFs and preparing the PIC for ribosomal subunit joining.
5. The last step in translation initiation is ribosomal subunit joining (binding of the large ribosomal subunit to the PIC), promoted by the GTPase eIF5B and eIF1A. eIF5B then hydrolyzes GTP and is released together with eIF1A, leaving behind a ribosome ready to translate the mRNA.

**Fig. 1:**
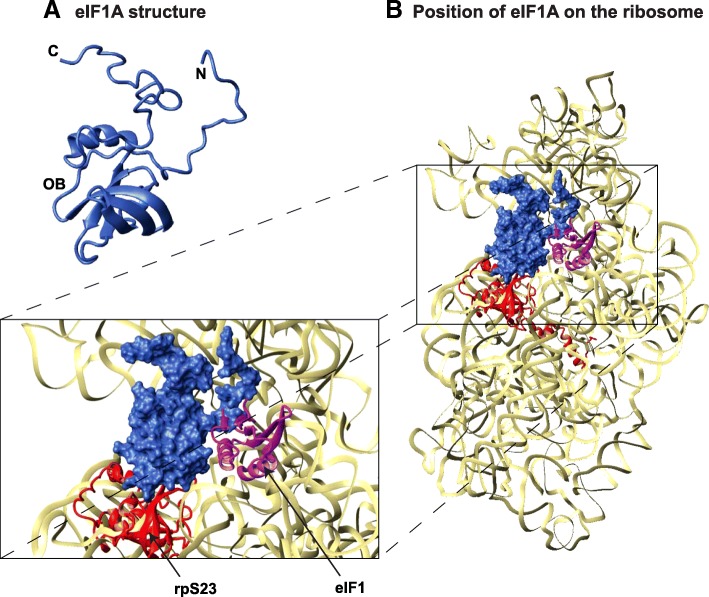
eIF1A structure and position on the ribosome. **a** eIF1A structure shown in ribbon. The OB-fold domain and the N- and C-termini are labeled. **b** Position of eIF1A, in surface representation, on the ribosome [36]. The 18S rRNA is shown in beige; eIF1 (magenta) and small ribosomal subunit protein 23 (rpS23, red) are shown and labeled in the zoomed-in box on the bottom-left. The rest of the small ribosomal subunit proteins are not shown. The Tetrahymena thermophila 40S ribosomal subunit and eIF1 are from 4bts.pdb; human eIF1A is from 1d7q.pdb

Translation initiation in bacteria is less complex, involving only three translation initiation factors (IFs), two of which, IF1 and IF2, are homologs of eIF1A and eIF5B, respectively. There is no scanning; instead, the small ribosomal subunit binds directly at the translation start site (reviewed in [3]). eIF1A is universally conserved in all Kingdoms of life. It shares with its bacterial homolog, IF1 the same binding site on the ribosome [5–8] and common functions. They both: (i) bind in the Aminoacyl-tRNA binding site (A-site) of the small ribosomal subunit and induce conformational changes in the ribosome, mimicking those caused by the binding of an aminoacyl-tRNA in the A-site [5, 7]; (ii) promote the assembly of the PIC at the start codon; and (iii) play a role in ribosomal subunit joining. Both IF1 and eIF1A have an oligonucleotide/oligosaccharide binding fold (OB) domain. eIF1A also has a helical subdomain, as well as N- and C-terminal tails (NTT and CTT, respectively) which are intrinsically disordered [9, 10] (Fig. 1a). eIF1A has acquired a number of eukaryote-specific functions and plays a role in virtually every step of the process of translation initiation. Together with other eIFs, eIF1A promotes PIC formation, mRNA binding, scanning, start codon selection, and ribosomal subunit joining. eIF1A has been reported to bind to several other eIFs: eIF2, eIF3, eIF5, and eIF5B; however, only the interaction interfaces with eIF5B have been mapped (reviewed in [1–4]). eIF1A and eIF1 were found to bind to the ribosome immediately adjacent to each other, although no productive interactions between the two proteins were observed [6, 7].

The first evidence for a second eIF1A homolog in eukaryotes, the eIF1A domain containing protein (eIF1AD), came from genome sequencing projects (see e.g. Human, *Schizosaccharomyces pombe (S. pombe), Caenorhabditis elegans (C. elegans)*). The protein has also been called Haponin in human [11, 12] and Obelix in chicken [13]. eIF1AD is typically annotated in databases as an RNA-binding protein and a translation initiation factor, owing to its homology to eIF1A; however, there is no available supporting experimental evidence for either. High-throughput expression, interaction and phenotype studies have provided limited information about the function of eIF1AD. Deletion of the gene in *S. pombe* caused abnormal cell shape, but showed that eIF1AD is not essential [14]. A number of alleles are reported in *C. elegans*, including an embryonic lethal (www.wormbase.org, gene ID ZK856.11), indicating that eIF1AD is essential in this organism. The protein was found to be preferentially localized to the nucleus in human [12], chicken [13], as well as *S. pombe* [15], which makes a role at least in canonical translation initiation unlikely. Yeast two-hybrid (Y2H) studies indicate that human eIF1AD interacts with the signal transducer and activator of transcription 1 (STAT1) transcription factor [16] and glyceraldehyde 3-phosphate dehydrogenase (GAPDH) [11]. The *C. elegans* eIF1AD homolog was reported to interact with ferritin heavy chains 1 and 2 in Y2H screens, while the *Drosophila melanogaster* (*D. melanogaster)* protein was found to bind to the transcription factor Extradenticle (Exd) [17, 18]. eIF1AD was found to be highly expressed in testes and ovaries in *C. elegans* and *Drosophila* (www.wormbase.org, gene ID ZK856.11; flybase.org, gene ID FBgn0051957) and upon neural induction in chicken [13]. Its overexpression in mammalian cells was reported to increase sensitivity to oxidative stress [12]. Thus, the available data fail to offer insights into the functions of eIF1AD. Recently, the Nuclear Magnetic Resonance (NMR) solution structure of human eIF1AD was solved by the Yokoyama group as part of the RIKEN Structural Genomics/Proteomics Initiative (2dgy.pdb). The structure shows significant similarity to the structure of its paralog eIF1A [9], as expected from the sequence homology between the two proteins. Like eIF1A, eIF1AD consists of a folded domain composed of an OB-fold and helical subdomains, flanked by intrinsically disordered N- and C-terminal tails.

eIF1AD is interesting in that its cellular function is unknown while at the same time its structure has been solved and it has a paralog (eIF1A) with extensively characterized functions and interactions. Based on the sequence and structure homology between eIF1A and eIF1AD, we reasoned that there may be conservation of the interaction surfaces of these two proteins. For example, eIF1A and eIF1AD could use the same surfaces for interactions with their respective ligands, or could even have a common interacting partner. If this is indeed the case, one can expect the corresponding surfaces to be conserved between the two proteins. Therefore, eIF1AD is a very promising candidate for applying bioinformatics sequence and structure analysis to generate hypotheses about its functions and interactions. In this work, we report remarkable conservation between the ribosome-binding surfaces of eIF1A and the corresponding regions in eIF1AD. These results indicate that eIF1AD may bind to the ribosome, similar to its paralog eIF1A, and could have roles in ribosome biogenesis or regulation of translation. We also identified potential protein-protein interaction motifs in eIF1AD. Our analysis of eIF1A identified a set of trypanosomatid-specific surface determinants that could be a promising target for development of treatments against these parasites.

The main goals of this work were to:

1. Identify regions on the eIF1AD surface with high degree of sequence conservation, since these are likely to be functionally important ligand-binding sites.
2. Compare surfaces conserved in eIF1AD with the corresponding regions in eIF1A. A surface conserved between the two proteins may serve the same function/bind to the same ligand.
3. Analyze the sequence conservation of regions in eIF1A and eIF1AD in individual branches of the eukaryotic domain. The goal was to obtain insights into whether any functions/interactions mapped to the respective region are conserved among all eukaryotes or are restricted to certain groups of organisms. This analysis allows determining when it is appropriate to extrapolate results obtained from one species to others. Conversely, it can also point out important functional differences between model organisms.

## Methods

### Sequence homology searches and sequence alignments

We used a non-redundant protein PSI-BLAST [19] tool from NCBI (http://blast.ncbi.nlm.nih.gov/Blast.cgi) with maximum target sequences set to 20,000. The results were then curated manually based on E-value and protein length, to eliminate incomplete sequences. We extracted representative sets of sequences from the alignments, based of pairwise sequence identity and minimum coverage using HHfilter [20, 21] from the Max-Planck Institute for Developmental Biology Bioinformatics Toolkit (http://toolkit.tuebingen.mpg.de). The sequences were aligned using ClustalW [22, 23] through the Max-Planck Institute for Developmental Biology Bioinformatics Toolkit (http://toolkit.tuebingen.mpg.de/). The alignment results were checked manually. ClustalW multiple sequence alignments and protein structures were used as input for ESPript [24] (http://espript.ibcp.fr) to produce sequence alignments color-coded for sequence conservation, also showing secondary structure elements and solvent accessibility.

### Protein structure analysis

We used Molmol [25] for structure analysis and visualization. For homology modeling of protein structures we used Swiss Model [26], in alignment mode. The sequence alignments were obtained using ClustalW [22, 23]. Sequence conservation was mapped onto the protein structures in Molmol, using Protskin [27] (http://www.mcgnmr.mcgill.ca/ProtSkin/). The consensus sequence as well as the conservation scores were both recorded. A threshold similarity score for conservation was selected based on the distribution of scores for the particular set of sequences as well as the percent identity.

## Results

As the first step in this work, we analyzed the sequence conservation of functionally important regions in eIF1A. The goals were twofold. The first one was to provide a reference point required for the subsequent analysis of eIF1AD. The second goal was to find out whether individual eIF1A interactions are conserved among all eukaryotes or only within certain branches of the eukaryotic domain, since this type of analysis has not been previously performed on eIF1A.

### Sequence conservation of surfaces in eIF1A

**Fig. 2:**
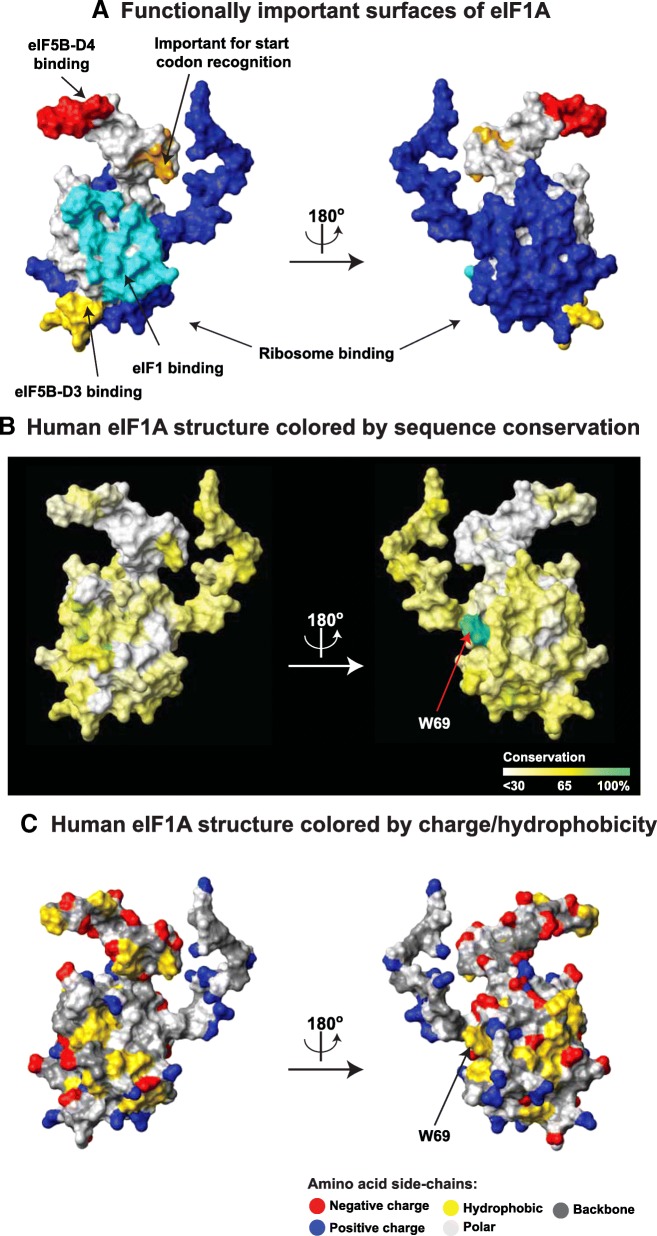
eIF1A interactions and surface conservation. **a** Functionally important surfaces of eIF1A. The human eIF1A structure in the same orientation as in Fig. 1 (left), and rotated 180 degrees (right). Interaction surfaces are color-coded and labeled. **b** Human eIF1A structure colored by sequence conservation. The eIF1A structure in the same orientation as in A, colored by sequence conservation. The universally conserved W69 is labeled. **c** Human eIF1A structure colored by charge/hydrophobicity

eIF1A is involved in a number of protein/protein and protein/RNA interactions [6–8, 28, 29]. Therefore, we set out to analyze whether each of the respective interaction surfaces in the protein is conserved. As described above, eIF1A is composed of a folded domain surrounded by an N-terminal and a C-terminal tails, which are intrinsically disordered (Fig. 1a). The folded domain itself consists of an OB-fold subdomain and a helical subdomain [9] (Fig. 1a). Extensive surfaces in the folded domain, as well as the NTT bind to the small 40S ribosomal subunit [6–8] (Fig. 1b, colored blue in Fig. 2a). On the 40S subunit, eIF1A also comes in close proximity to eIF1 (Fig. 1b, the corresponding eIF1A surface is colored cyan in Fig. 2a); however there are no obvious productive interactions [6, 7]. The extreme C-terminus of eIF1A binds to the C-terminal domain of eIF5B (eIF5B-CTD) [28, 29] (red in Fig. 2a). Another segment of eIF1A-CTT, closer to the folded domain (orange in Fig. 2a), plays a role in maintaining the stringency of start codon recognition [30], but it is not clear whether this is mediated by protein/protein interactions. The folded domain of eIF1A also contacts eIF5B (gold in Fig. 2a) [6–8, 31, 32]. The remaining surfaces of eIF1A (grey in Fig. 2a) were considered a separate group in the analysis.

**Fig. 3:**
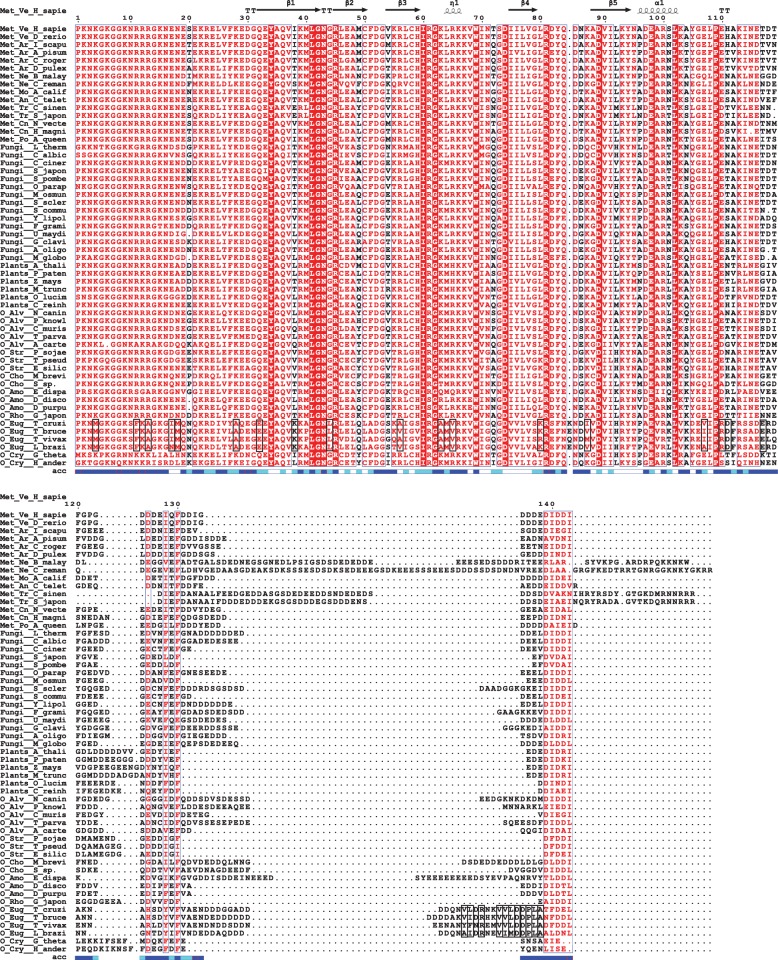
Multiple sequence alignment of eIF1A sequences. Residue numbers and secondary structure elements for human eIF1A are shown above the alignment. Solvent accessibility is shown below the alignment. Residues in the sequences from Trypanosomatida species (Phylum Euglenozoa) showing divergence from the rest of the eIF1A sequences are boxed. Abbreviations: Met, Metazoa; O, others; Ve, Vertebrates; Ar, Arthropoda; Ne, Nematoda; Mo, Mollusca; An, Annelida; Tr, Trematoda; Cn, Cnidaria; Po, Porifera; Alv, Alveolata; Str, Stramenopiles; Cho, Choanoflagellida; Amo, Amoebozoa; Rho, Rhodophyta; Eug, Euglenozoa; Cry, Cryptophyta

The eIF1A sequence conservation is very high: nearly identical among all mammals; > 80% identity among vertebrates, with the zebra fish (*Danio rerio*) sequence, for instance, being 99% identical to that of human eIF1A. Even the sequence and length of the intrinsically disordered tails are conserved. Comparisons among the different eukaryotic kingdoms show that the NTT and the folded domain of eIF1A remain well conserved, whereas the sequence and length of the CTT are less conserved (Fig. 3, Fig. 2b). For example, human and *Saccharomyces cerevisiae (S. cerevisiae*) eIF1A sequences have 62% identity overall and 69% identity with no gaps over the NTT and the folded domain (excluding the CTT).

As expected, the ribosome-binding surfaces are most conserved, with clear conservation of positively charged residues (compare Fig. 2a, b, and c). By far the most conserved surface-exposed residue is the invariant W69 (Fig. 3, labeled in Fig. 2b, c), at the ribosome binding surface. The eIF1A surface facing eIF1 is least conserved (compare Fig. 2a, b, and c).

Overall, we did not observe differential conservation in individual branches of the eukaryotic lineage (Fig. 3), with the two notable exceptions discussed below.

In roundworms (Phylum *Nematoda*) and flatworms (Phylum *Platyhelminthes*), the eIF1A C-terminus has no discernible eIF5B-CTD binding motif and carries a positive charge. Nematodes and Platyhelminthes belong to different clades: Ecdysozoans and Lophotrochozoans, respectively. eIF1A sequences from species belonging to other phyla from both of these clades, e.g. *Arthropoda* (Ecdysozoans) or *Annelida* and *Mollusca* (Lophotrochozoans) have a conserved eIF5B-CTD binding site and the entire eIF1A-CTT is negatively charged (Fig. 3). Therefore, the loss of the eIF5B-CTD binding site and the added positive charges must have occurred twice in evolution.

**Fig. 4:**
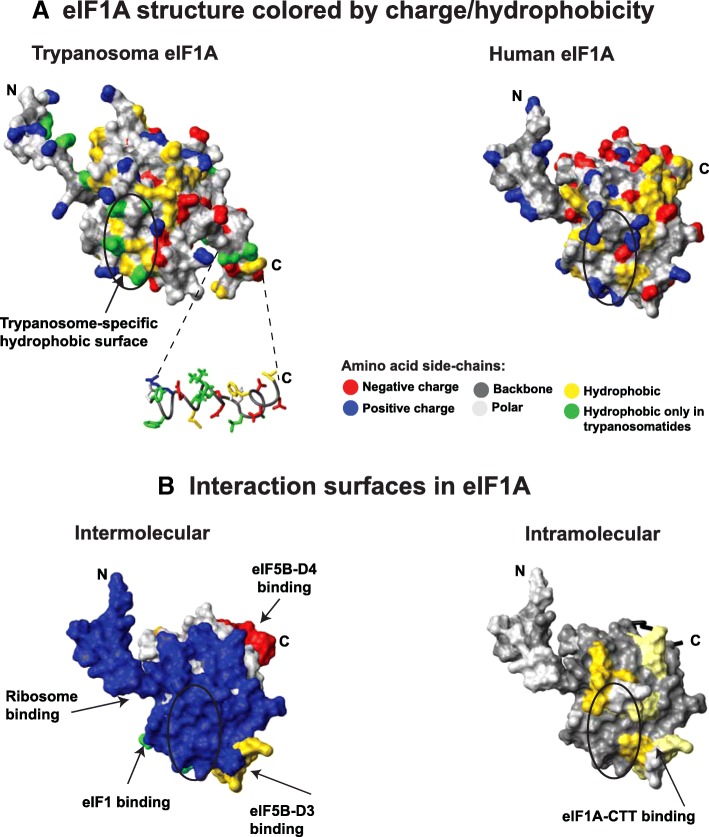
Unique features of trypanosoma eIF1A. **a** Homology model of *Trypanosoma vivax* eIF1A (left) and human eIF1A structure (right) colored by charge/hydrophobicity. The structures are in the same orientation as in Fig. 1. Trypanosoma-specific hydrophobic residues are shown in green; a unique hydrophobic surface is circled; and a hydrophobic segment in the C-terminus of *T. vivax* eIF1A is shown in ribbon as an inset in the left panel. **b** Functionally important surfaces of eIF1A are shown as a reference. Intermolecular interaction surfaces (left) are color-coded and labeled as in Fig. 2a. The intermolecular contact surface for eIF1A-CTT is shown on the right panel. The area corresponding to the Trypanosoma-specific hydrophobic surface is circled as in panel **a**

In trypanosomatids, eIF1A shows markedly different sequence conservation pattern, compared to any other group of organisms (Fig. 3, Fig. 4a). There is a trypanosomatid-specific area with substantial hydrophobicity located on the ribosome-binding surface (compare the circled region on the *Trypanosoma vivax (T. vivax)* eIF1A structure, Fig. 4a, left, with the corresponding region in human eIF1A, Fig. 4a, right, and Fig. 4b, left). The eIF1A C-terminus also has a segment with high hydrophobicity unique to trypanosomatids (Fig. 3, Fig. 4a, left).

### Homology between eIF1A and eIF1AD

eIF1AD is present only in eukaryotes. It must have been present in the last common ancestor of all eukaryotes, because some eukaryotes that have branched out early, e.g. *Giardia theta*, have an eIF1AD gene. At the same time, eIF1AD has been lost in a number of eukaryotes, including *S. cerevisiae*.

**Fig. 5:**
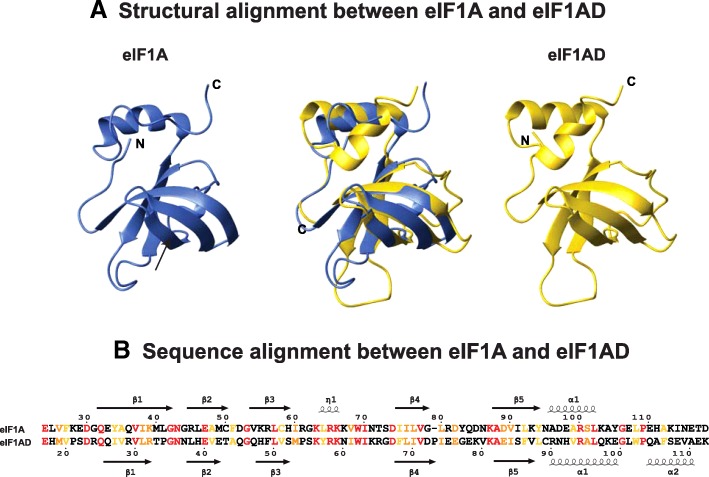
Comparison between eIF1A and eIF1AD. **a** Center, structure alignment between the folded domains of human eIF1A (1d7q.pdb, blue) and eIF1AD (2dgy.pdb, gold). The eIF1A and eIF1AD structures are shown on the left and right, respectively. **b** Sequence alignment of the folded domains of eIF1A (top) and eIF1AD (bottom). Identical residues are red; similar residues are orange; and conserved hydrophobic residues are gold. Residue numbers and secondary structure elements for human eIF1A are shown above the alignment. Residue numbers and secondary structure elements for human eIF1AD are shown below the alignment

**Fig. 6:**
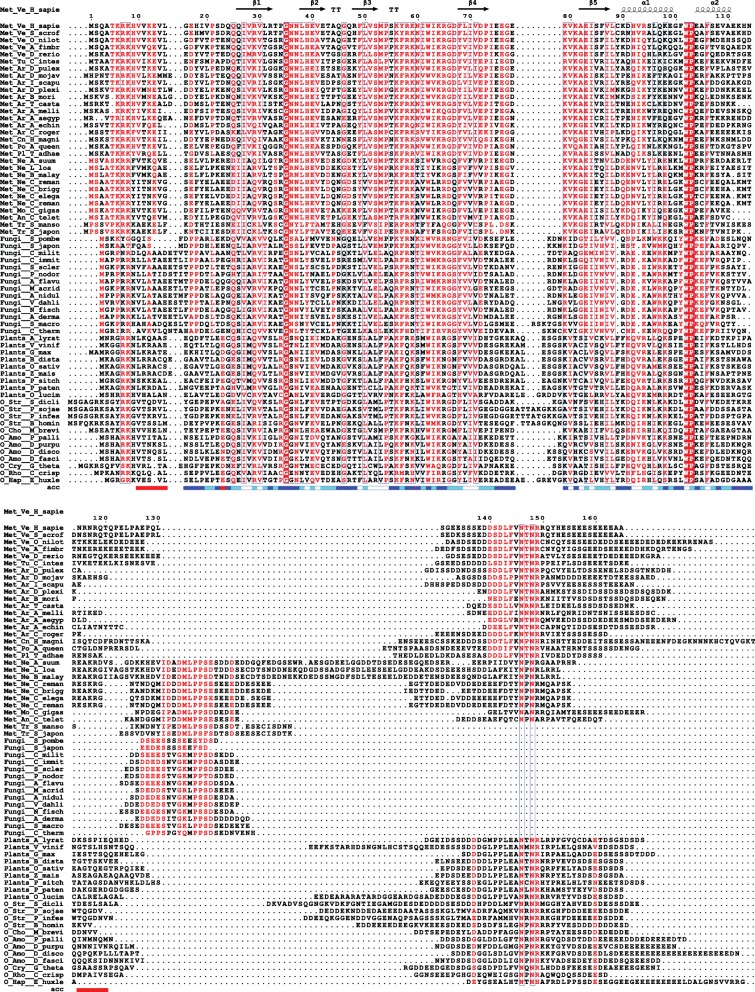
Multiple sequence alignment of eIF1AD sequences. Residue numbers and secondary structure elements for human eIF1AD are shown above the alignment. Solvent accessibility is shown below the alignment. Abbreviations: Met, Metazoa; O, others; Ve, Vertebrates; Tu, Tunicata; Ar, Arthropoda; Cn, Cnidaria; Po, Porifera; Pl, Platyhelminthes; Ne, Nematoda; Mo, Mollusca; An, Annelida; Tr, Trematoda; Str, Stramenopiles; Cho, Choanoflagellida; Amo, Amoebozoa; Cry, Cryptophyta; Rho, Rhodophyta; Hap, Haptophyta

Like eIF1A, eIF1AD has an OB domain surrounded by two intrinsically disordered tails (Fig. 5a). The sequence homology between the two proteins is highest in the folded domain with 23% identity and 34% homology (Fig. 5b). While the NTT sequences are not well conserved, they both have an overall positive charge. No similarity exists between the CTT sequences of eIF1A and eIF1AD (compare Figs. 3 and 6).

**Fig. 7:**
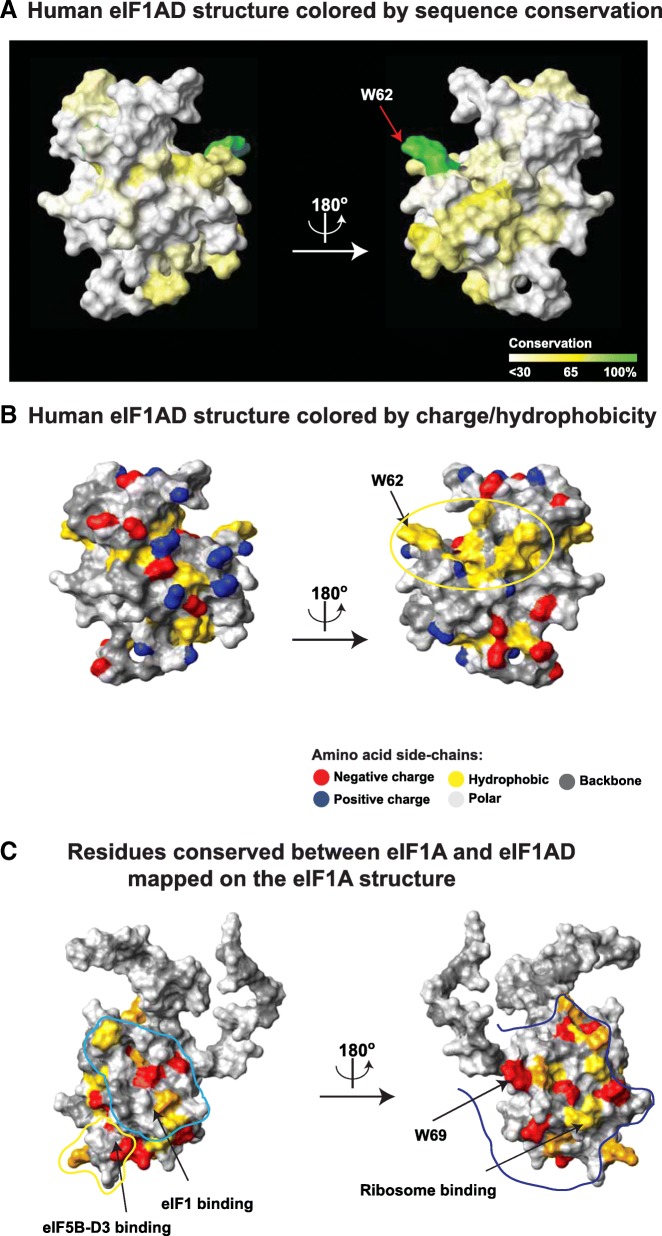
Surface conservation in eIF1AD and comparison with eIF1A. **a** Human eIF1AD structure colored by sequence conservation. The universally conserved W62 is labeled. **b** Human eIF1AD structure colored by charge/hydrophobicity. Functionally important surfaces of eIF1A. **c** Residues conserved between human eIF1A and eIF1AD (see Fig. 5b) mapped on the eIF1A structure in the same orientations as in panel A. Known interaction surfaces in eIF1A (see Fig. 2a) are outlined and labeled

The eIF1AD sequence conservation (Fig. 6) is not as high as that of eIF1A (see Fig. 2): > 80% identity among mammals; > 50% identity among vertebrates. As with eIF1A, the NTT and the folded domain of eIF1AD are well conserved, whereas the sequence and length of the CTT are not (Fig. 6). Analysis of surface-exposed residues in eIF1AD shows that the surfaces corresponding to ribosome-binding surfaces in eIF1A are among the best-conserved, with a substantial number of positively charged residues (Fig. 7a, b, compare with Fig. 2).

The folded domain of eIF1AD has a conserved surface with significant hydrophobicity (circled in Fig. 7b, right), which corresponds to part of the ribosome-binding surface in eIF1A, but is less conserved. This surface is a likely site of protein-protein interactions, since solvent-exposed hydrophobic residues are both energetically unfavorable and can contribute to stability and specificity of interactions. If eIF1AD does indeed bind to the ribosome in the same way as eIF1A, this surface would become buried at the interface.

While the eIF1AD C-terminal tail as a whole is not conserved, it contains highly conserved sequence motifs, which are likely protein-protein interactions sites (Fig. 6). One of the conserved motifs is found in most eukaryotes, but absent in Fungi and one branch of Metazoans: members of the phylum *Platyhelminthes*. We designate this motif here as “NTNR” (Asn-Thr-Asn-Arg), after the most conserved core of residues. The second motif is found in Fungi and the phyla *Platyhelminthes*, *Mollusca*, *Anelida*, and *Nematoda*. We designate this motif here as “LPPS” (Leu-Pro-Pro-Ser), after the most conserved core of residues. eIF1AD from *Mollusca*, *Anelida*, and *Nematoda* has both motifs (Fig. 6), which indicates that these are two independent sequence motifs, with independent functions. In plants, the eIF1AD-CTT contains an extended NTNR sequence motif, where the first portion of the sequence resembles the LPPS motif. It is thus possible that this motif has resulted from merging the NTNR and LPPS motifs in tandem in the plant eIF1AD sequence. Thus, the C-terminal tail of eIF1AD contains a set of sequence motifs that vary in consensus, and likely also function, among different branches of eukaryotes.

## Discussion

eIF1A is one of only two universally conserved translation initiation factors, found in every organism, from bacteria to human. There is also very high degree of conservation in eIF1A sequences among eukaryotes (Fig. 3). The invariant W69, at the ribosome binding surface (Fig. 3, labeled in Fig. 2b, c), is also conserved in the archaeal eIF1A homolog aIF1A, whereas the bacterial homolog IF1 has an arginine at this position (*not shown*). Remarkably, W69 was recently found to form a stacking interaction with a functionally important base A1709 in the Tetrahymena 18S small ribosomal RNA (rRNA), stabilizing it in a flipped-out conformation [7]. A1709 in helix 44 of the *Tetrahymena* 18S rRNA (A1819 in rabbit) corresponds to A1493 in *Escherichia coli* (*E. coli)*, which in its flipped-out form “inspects” the proper codon-anticodon basepairing in the Aminoacyl-tRNA site (A-site) of the ribosome [33]. During translation initiation in bacteria, A1493 is also flipped out by the bacterial eIF1A homolog IF1, thus inducing conformational changes in the ribosome that can mimic the presence of a tRNA in the A-site [5]. A W69A mutant was found to cause a defect in start codon recognition (48S initiation complex formation) in vitro and appearance of aberrant 48S complexes with mRNA not positioned correctly on the ribosome [9]. The defect in 48S complex formation was not as drastic as could have been expected from the exceptionally high degree of conservation of W69 and its observed interaction with A1709, and the mutation had little effect on the assembly of the pre-initiation complex off mRNA (43S complex formation) [9]. Therefore, it appears that the main role of W69 is to induce conformational changes in the 40S ribosomal subunit, rather than in eIF1A binding to the ribosome per se.

The eIF1A surface facing eIF1 is least conserved (compare Fig. 2a, b, and c), consistent with the observation that there are no productive contacts between the two proteins in the 40S/eIF1A/eIF1 crystal structure [6, 7]. This indicates that the observed ~ 10-fold cooperativity of eIF1A and eIF1 binding to the 40S ribosomal subunit [34] is likely mediated by both proteins promoting similar conformational changes in the ribosome. It is also possible that direct contacts between eIF1 and eIF1A do contribute to the cooperativity, since binding to the ribosome places eIF1A and eIF1 in such close proximity that even weak interactions between the two proteins could have a stabilizing effect.

The eIF5B-CTD binding motif at the eIF1A C-terminus is conserved in almost all eukaryotic species, except roundworms (Phylum *Nematoda*) and flatworms (Phylum *Platyhelminthes*), where the eIF1A-CTT carries a positive charge, instead (Fig. 3). The interaction between eIF1A-CTT and eIF5B-CTD was found to be important for ribosomal subunit joining in *S. cerevisiae* [35]. It is thus interesting to know whether eIF1A-CTT still plays the same role in these worms. There are no compensatory changes in the respective surface on eIF5B-CTD (*not shown*); therefore, it is highly unlikely that the eIF1A-CTT can still bind there. Alternatively, since eIF5B-CTD interacts with the large ribosomal subunit, the positive charge of eIF1A in these species could allow it to bind to the rRNA in the vicinity of eIF5B-CTD.

As described above (Fig. 3), trypanosomatid eIF1A sequences appear to have diverged from the consensus in the rest of eukaryotes. Regions with increased hydrophobicity, conserved among trypanosomatids, but not other species, are observed in the NTT, CTT, and certain surfaces of the folded domain (Fig. 3, Fig. 4a). These regions may be sites of novel trypanosomatid-specific interactions. While trypanosomatid translation initiation is still not fully understood, there are a number of known differences from other eukaryotes. We reported recently that the eIF1A C-terminus dynamically contacts the ribosome-binding surface of eIF1A, an interaction that is disrupted when eIF1A binds to the ribosome ([36], Fig. 4b, right). Therefore, the two trypanosomatid-specific hydrophobic segments likely contact each other when eIF1A is not ribosome-bound. However, once eIF1A is bound to the ribosome, both its NTT and CTT are free to interact with other proteins [8, 30]. eIF1A interacts with eIF5B via regions adjacent to the trypanosomatid-specific hydrophobic surfaces [28, 29, 36] (see also Fig. 2a). eIF1A is known to interact with eIF2, eIF3 and the C-terminal domain of eIF5 (eIF5-CTD) [29, 37, 38], and on the ribosome, both eIF1A-NTT and -CTT are in proximity to eIF2, eIF3c, and eIF5, as well as to eIF1 [8, 30, 38–40]. We did not observe any trypanosomatid-specific hydrophobic surfaces in eIF1 or eIF5B (data not shown). Therefore, it is unlikely that the trypanosomatid-specific hydrophobic surfaces in eIF1A affect the interactions with eIF1 or eIF5B. The degree of sequence conservation of eIF3c and eIF5-CTD is too low for meaningful analysis and we cannot make any predictions about their interactions with eIF1A. Two of the three eIF2 subunits, α and β, have trypanosomatid-specific characteristics: eIF2α has a small N-terminal domain not found in most other eukaryotes, while eIF2β-NTT lacks the three conserved K-boxes that in other eukaryotes bind eIF5-CTD (see above). Thus, while the functional significance of these differences is not known, both eIF2α and -β could interact with the unique hydrophobic surfaces in trypanosomatid eIF1A. It is of course possible that the trypanosomatid-specific surfaces in eIF1A form novel interactions unique to trypanosomatids. For instance, trypanosomatid mRNAs are first transcribed as large polycistronic mRNAs and their maturation involves trans-splicing, adding a ~ 40 nt capped leader sequence to every mRNA. Trypanosomatids contain multiple eIF4E and eIF4G isoforms [41–46]. It was recently reported that in mammals, the PIC inspects the mRNA from the very 5′-end, placing the cap-binding complex in the vicinity of the ribosomal A- and P-sites at the beginning of scanning [47]. If this is also the case in trypanosomatids, then eIF1A could also be involved in trypanosomatid-specific interactions with eIF4A, 4E, and/or 4G. Since a number of trypanosomatids are parasites, a unique hydrophobic region on the ribosome-binding surface of an essential protein like eIF1A is a promising therapeutic target.

While eIF1AD is present in most eukaryotes, its sequence conservation s not as high as that of eIF1A (Fig. 6). Nevertheless, there is significant conservation not only among eIF1AD sequences, but also between eIF1A and eIF1AD (Fig. 5b). The majority of surface-exposed residues conserved between eIF1A and eIF1AD map to the ribosome-binding surface of eIF1A (Fig. 7c). The most conserved surface-exposed residue in eIF1AD, the almost invariant W62 (labeled in Fig. 7) also maps to the ribosome-binding surface. Remarkably, eIF1AD W62 corresponds to the most conserved residue in eIF1A, W69 (Fig. 2, Fig. 3b). As discussed above, W69 is involved in promoting conformational changes in the ribosome upon eIF1A binding [7]. The significant sequence conservation between the ribosome-binding surfaces of eIF1A and the corresponding regions of eIF1AD indicates that eIF1AD is also likely to bind to the ribosome, or rRNA. This hypothesis is further strengthened by the observation that the same tryptophan residue known to be important for eIF1A function is the most conserved surface-exposed residue in both proteins. Furthermore, the high degree of sequence conservation in the positively charged eIF1AD-NTT indicates that it may also be involved in ribosome binding, similar to eIF1A-NTT. If this is indeed the case, the function of such eIF1AD interaction remains to be determined. The eIF5B-binding regions of eIF1A are not conserved with eIF1AD, indicating that eIF1AD does not interact with eIF5B. Therefore, eIF1AD either does not act as an alternative translation initiation factor or it functions in a unique pathway that does not involve eIF5B. A number of homologs of canonical translation initiation factors have been described. In most, if not all, of the cases where the functions of these homologs are known, they are in some way or other related to translation. For example, Pdcd4, PAIP1, 5MP1, and 5MP2, which are homologs of eIF4G and eIF5, are translation regulators [48–51]. The nuclear Cap-Binding Complex, composed of CBP80 (an eIF4G homolog) and CBP20 (homologous to eIF4B and 4H) [52] plays multiple roles in transcription, mRNA maturation and export, as well as the pioneer round of translation and Nonsense-Mediated Decay (NMD) [53, 54]. eIF4A3 (a homolog of eIF4A) is part of the Exon Junction Complex and is involved in mRNA export and NMD [54–56], as well as in rRNA biogenesis, together with another eIF4G homolog, NOM1 [57]. Therefore, eIF1AD is likely involved in one or more of these processes. Since eIF1AD has been found to be localized predominantly in the nucleus [12, 13, 15], direct role in translation regulation is somewhat less likely. Instead, it could play roles in regulation of ribosome biogenesis or mRNA maturation.

## Conclusions

In summary, our structure/sequence analysis of eIF1AD found significant conservation in the surfaces corresponding to the ribosome-binding surfaces of its paralog eIF1A. Remarkably, both protein families share a nearly invariant surface-exposed tryptophan residue, which plays an important role in the interaction of eIF1A with the ribosome. These results indicate that eIF1AD may bind to the ribosome, similar to its paralog eIF1A, and could have roles in ribosome biogenenesis or regulation of translation. We also identified conserved surfaces and sequence motifs in the folded domain as well as the CTT of eIF1AD, which are likely protein-protein interaction sites. The roles of these regions for eIF1AD function remain to be determined. Furthermore, our analysis of eIF1A identified a set of trypanosomatid-specific surface determinants that could be a promising target for development of treatments against these parasites. We expect that the results and hypotheses described here will promote new research and help elucidate the functions of eIF1AD.

## Abbreviations

A-site: Aminoacyl-tRNA site
*C. elegans*: *Caenorhabditis elegans*
CTD: C-terminal domain
CTT: C-terminal tail
*D. melanogaster*: *Drosophila melanogaster*
*E. coli*: *Escherichia coli*
eIF1A: Eukaryotic translation initiation factor 1A
eIF1AD: eIF1A domain-containing protein
*Exd*: Extradenticle
GAPDH: Glyceraldehyde 3-phosphate dehydrogenase
IF1: Initiation factor 1
NMD: Nonsense-Mediated Decay
NMR: Nuclear Magnetic Resonance
NTT: N-terminal tail
OB: Oligonucleotide/oligosaccharide binding fold
rpS: Small ribosomal subunit protein
rRNA: ribosomal RNA
*S. cerevisiae*: *Saccharomyces cerevisiae*
*S. pombe*: *Schizosaccharomyces pombe*
STAT1: Signal transducer and activator of transcription 1
*T. vivax*: *Trypanosoma vivax*
Y2H: Yeast two-hybrid
